# Characterization of multidrug-resistant ***Acinetobacter ssp.*** strains isolated from medical intensive care units in Cali - Colombia.

**DOI:** 10.25100/cm.v48i4.2858

**Published:** 2017-12-30

**Authors:** Rómel Fabian Gómez, Andres Castillo, Mónica Chávez-Vivas

**Affiliations:** 1 Grupo de Investigación en Microbiología Molecular y Enfermedades Infecciosas (GIMMEIN). Universidad Libre, seccional Cali. Colombia.; 2 Departamento de Biología. Facultad de Ciencias Naturales y Exactas. Universidad del Valle, Cali. Colombia.; 3 Grupo Microambiente Libre Departamento de Ciencias Biomédicas. Facultad de Salud. Universidad Santiago de Cali. Cali. Colombia.; 4 Grupo de Investigación Instituto de Ciencias Biomédicas. Universidad Libre de Cali, Colombia.

**Keywords:** Acinetobacter Infections, Multiple Drug Resistance, 16S Ribosomal RNA, Healthcare Associated Infections, Infecciones, Acinetobacter, resistencia, medicamentos, ARN ribosomal 16S, cuidado de la salud.

## Abstract

**Introduction:**

The extensive use of antibiotics has led to the emergence of multi-resistant strains in some species of the genus *Acinetobacter*.

**Objective:**

To investigate the molecular characteristics of multidrug-resistant of *Acinetobacter ssp.* strains isolated from 52 patients collected between March 2009 and July 2010 in medical intensive care units in Cali - Colombia.

**Methods:**

The susceptibility to various classes of antibiotics was determined by disc diffusion method, and the determination of the genomic species was carried out using amplified ribosomal DNA restriction analysis (ARDRA) and by sequencing of the 16s rDNA gene. Also, the genes of beta-lactamases as well as, integrases IntI1 and IntI2 were analyzed by PCR method.

**Results:**

The phenotypic identification showed that the isolates belong mainly to *A. calcoaceticus- A. baumannii complex.* All of them were multi-resistant to almost the whole antibiotics except to tigecycline and sulperazon, and they were grouped into five (I to V) different antibiotypes, being the antibiotype I the most common (50.0%). The percent of beta-lactamases detected was: blaTEM (17.3%), blaCTX-M (9.6%), blaVIM (21.2%), blaIMP (7.7%), blaOXA-58 (21.2%), and blaOXA-51 (21.2%). The phylogenetic tree analysis showed that the isolates were clustering to *A. baumannii* (74.1%), *A. nosocomialis* (11.1%) and *A. calcoaceticus* (7.4 %). Besides, the integron class 1 and class 2 were detected in 23.1% and 17.3% respectively.

**Conclusion:**

The isolates were identified to species *A. baumanii* mainly, and they were multiresistant. The resistance to beta-lactams may be by for presence of beta-lactamases in the majority of the isolates.

## Resumen

**Introducción:**

El uso extensivo de antibióticos ha llevado a la emergencia de cepas multirresistentes en algunas especies del género *Acinetobacter*.

**Objetivo:**

Investigar las características moleculares de resistencia a múltiples fármacos de cepas aisladas de *Acinetobacter spp*. colectadas entre marzo de 2009 y julio de 2010 en 52 pacientes de unidades de cuidados intensivos en Cali - Colombia.

**Métodos:**

La susceptibilidad a diversas clases de antibióticos se determinó mediante el método de difusión de disco, y la determinación de la especie genómica se llevó a cabo usando un análisis de restricción de ADN ribosómico amplificado (ARDRA) y mediante la secuenciación del gen 16s de ADNr. Además, se analizaron por el método de PCR los genes de las beta-lactamasas, como también, las integrasas IntI1 e IntI2.

**Resultados:**

La identificación fenotípica mostró que los aislamientos pertenecen principalmente al complejo *A. calcoaceticus* - *A. baumannii*. Todos ellos eran multirresistentes a casi todos los antibióticos excepto tigeciclina y sulperazón, y se agruparon en cinco (I a V) antibitipos diferentes, siendo el antibiotipo I el más común (50%). El porcentaje de betalactamasas detectadas fue: *bla*TEM (17,3%), *bla*CTX-M (9,6%), *bla*VIM (21,2%), *bla*IMP (7,7%), *bla*OXA*-58* (21,2%), *bla*OXA*- 51* (21.2%). El análisis del árbol filogenético mostró que los aislados se agrupaban en *A. baumannii* (74.1%), *A. nosocomialis* (11.1%) y *A. calcoaceticus* (7.4%). Además, el integrón clase 1 y clase 2 se detectaron en 23.1% y 17.3% respectivamente.

**Conclusión:**

los aislamientos se identificaron principalmente como la especie *A. baumanii*, y fueron multirresistentes. La resistencia a los betalactámicos puede deberse a la presencia de betalactamasas en la mayoría de los aislamientos.

## Introduction

*Acinetobacter spp*. is reported to be involved in hospital-acquired infections with increasing frequency ^1^. The extensive use of antimicrobial chemotherapy in clinical environments has contributed to the emergence and dissemination of nosocomial *Acinetobacter spp* infections ^2^. The species belonging to the *A. calcoaceticus-A. baumannii* complex (ACB complex) is mostly antibiotic-resistant Acinetobacter strains ^3^. This complex has been implicated as the cause of a broad spectrum of infectious diseases such as pneumonia, meningitis, bacteremia, urinary tract infections, and device-related infections, especially in intensive care units (ICUs), and high mortality rates were associated ^4^. Due to the organism's multidrug-resistant (MDR) phenotype, these infections are difficult to treat, which includes resistance to beta-lactams, aminoglycosides, fluoroquinolones, and carbapenems ^5^ ^,^ ^6^. A significant nosocomial outbreak of MDR ACB complex in Colombia has occurred in the last years ^7^.

Resistance to beta-lactam agents, including carbapenems in the ACB complex is due mainly to the production of the extended-spectrum beta-lactamases (ESBLs), but can also result from several other mechanisms including alterations in outer membrane proteins and penicillin binding proteins and increased activity of efflux pumps ^8^. ESBLs with carbapenemases, such as metallo-beta-lactamases (MBL) or oxacillinases, represent the most concern due to the chance of rapid dissemination ^9^. Whereas MBL found are of IMP and VIM types in *A. baumannii* ^5^, the oxacillinases present four main OXA subgroups are the chromosomally located intrinsic OXA-51-like; the acquired OXA-23-like; OXA-40-like; and OXA-58-like ^9^ ^-^ ^12^. 

Most genes encoding these ESBLs are found on plasmids, or in the form of a gene cassette in an integron. Integrons are genetic elements that possess a particular recombination site, known as *attI1*, into which resistance genes can be inserted by site-specific recombination in the form of gene cassettes ^13^. Different integron classes have been described, and the classes 1, 2 and 3 have been associated with antibiotic resistance ^13^ ^,^ ^14^. Dissemination of these antibiotic resistance integrons, which are unable to promote their mobilization, is mainly linked to transposons and plasmids. Different reports exist which have identified integrons as responsible for the presence and acquisition of antibiotic resistance *A. baumannii* have been published ^13^ ^,^ ^14^. There is limited data on the global epidemiology of *A. baumannii* in our region. However, the distribution of class 2 integron is highly frequent in *A. baumannii* clinical isolates from Argentina, Chile, and Brazil ^14^ ^,^ ^15^. 

The objectives of the present study were to analyze the phenotypic and molecular characteristics of ACB complex and antibiotic resistance in clinical isolates in a Colombian tertiary-care hospital. As well as determine the antimicrobials resistance profiles of the isolated strains. We also tested for the presence of MBL or ESBL producing *Acinetobacter* spp. 

To test the hypothesis that most genes encoding MBL or ESBL are found on gene cassettes of integrons, we have examined representative isolates for the presence of class 1 and class 2 integrons and the associated antimicrobial resistance genes by PCR. Knowledge on the epidemiology and molecular mechanisms of antimicrobial resistance in this important pathogen are essential to implement intervention strategies.

## Material and Methods 

A descriptive study was carried out and was endorsed by the ethics committee of the hospital.

### Bacterial strains clinical samples

The study included 52 *Acinetobacter* ssp strains collected from clinical specimens, previously identified by the Vitek GNI card (bioMeriex Vitek Inc., Hazelwood, MO) that they grew on standard MacConkey agar at 37° C for 48 h, between March 2009 and July 2010 of a medical intensive care unit at Rafael Uribe Uribe University Hospital from Cali - Colombia. The clinical samples were obtained from: the nasal swabs, 24 (46.2%); wounds, 12 (26.1%); catheter tip, 6 (11.6%); the urinary tract, 6 (11.6%); and 4 (7.7%) blood. All of the samples were stored at −80° C in nutrient broth with 15% glycerol. From the 52 *Acinetobacter* spp clinical isolates strains, 24 (46.2%) were obtained. 

### Antimicrobial susceptibility testing

The susceptibility to various classes of antibiotics was determined by disc diffusion method on a Mueller-Hinton (MH) agar (Difco Laboratories, Detroit, MI, USA), by the Clinical and Laboratory Standard Institute (CLSI) guidelines ^16^. Both strains *Acinetobacter baumannii* (ATCC® 19606(tm)), and *Escherichia coli* (ATCC® 25922(tm)) were used as quality control strains. The antibiotic discs (Oxoid ®) tested were the follows: trimetroprim/sulfametoxazol (SXT, 23.75 μg/1.25 μg); ticarcillin/clavulanato (TIM, 75μg/10 μg); gentamicin (GEN, 10 μg); tobramycin (TOB, 10 μg); ciprofloxacin (CIP, 5 μg); ceftazidime (CAZ, 30 μg); cefepime (FEP, 30 μg); aztreonam (ATM, 30 μg); imipenem (IMP, 10 μg); meropenem (MEM, 10 μg); ampicillin/sulbactam (SAM, 10 μg/10 μg); amikacin (AMK, 10 μg); cefoprazone/sulbactam (sulperazona, SUL, 75 μg/30 μg); and tigecycline (TIG, 15 μg). According to Manchanda et al. ^17^, the MDR *Acinetobacter spp* have defined whether the isolates are resistant to three antibiotics different to ceftazidime, such as ciprofloxacin, gentamicin, and imipenem.

### Identification of isolates of Acinetobacter spp: Amplified Ribosomal DNA Restriction Analysis (ARDRA)

The genomic species of *Acinetobacter spp* were determined by ARDRA, according to Vaneechoutte et al. ^18^. In brief, the DNA from an overnight culture in LB broth at 37° C was extracted using the "Easy-DNA^TM^" kit (Invitrogen, life technologies ®) according to the manufacturer's instructions. After to electrophoresis, the gel was stained with ethidium bromide (1.5 μg/mL) and analyzed on a FOTO/Analyst(r) Investigator/FX Systems (FOTODYNE Incorporated). For each sample, the PCR reaction was performed for 16S rDNA amplification using the following primer pair: *Acf*5´-TGG CTC AGA TTG AAC GCT GGC GGC-3´ and *Acr*5*´-*TAC CTT GTT ACG ACT TCA CCC CA-3´. The PCR products were digested with the following enzymes: *CfoI; Alu; Mbo;* and *MspI* (Fermentas(tm)) and separated by polyacrylamide gel electrophoresis (PAGE 8.0%). 

The last restriction profiles were compared to strain library database (http://users.ugent.be/~mvaneech/ARDRA/Acinetobacter.html) to identify the species according to Dijkshoorn *et al* ^19^. The *A. baumannii* (ATCC® 19606(tm)) strain was used as controls. 

### Identification of clinical isolates of Acinetobacter spp by 16S rDNA PCR-sequencing

The 27 PCR products to 16S rDNA gene were purified using the High Pure PCR product Purification Kit Version 20 kit, according to company instruction (Roche Applied Science®) and then, each purified PCR products were direct sequencing by Sanger dideoxy method using an ABI 3730XL sequencer. The 16S rDNA sequences were aligned with 27 Acinetobacter reference sequences harbored in the GenBank-NCBI dataset. 

The phylogenetic tree was done using the MEGA software v.5 under Maximum Likelihood model with the Kimura-2-parameters, and Gamma distribution assuming invariable sites (K2+G+I). The robustness of phylogenetic tree was calculated by a bootstrap no parametric with 1,000 replicates ^20^ ^,^ ^21^. A *Staphylococcus aureus* sequence was used as outgroup.

### Detection of ESBL genes by PCR

ESBL genes, such as *blaTEM; blaSHV; blaOXA; blaCTX-M; blaIMP; and blaVIM*, were detected by PCR using the primers are listed in Table 1 ^22^ ^-^ ^30^ ^).^ The PCR reaction final volume of 50 μl with 5-10 ng (genomic DNA) reaction buffer, 1 U of Taq polymerase (Bioline, London, United Kingdom), 200 µM each deoxynucleoside triphosphate, 1.5 or 2.5 mM MgCl_2_, 10 pmol of each primer. Thermocycler temperature was 94° C for 5 min; 94° C for 30 s by 35 cycles. To *blaTEM,* 52° C for 45 s; *blaOXA-51 and blaOXA-58,* 62° C for 1 min; *blaCXT-M,* 51° C for 45 s; *blaVIM and blaIMP* 51° C for 1 min; and 72° C for 60 s. The final step of 10 min at 72° C.

**Table 1:** Primers used in the study

Primer	Oligonucleotide sequences	Size (bp)	Reference
rDNA16S AC	F-5'_TGGCTCAGATTGAACGCTGGCGGC_3'	1,500	18
	R-5'_TACCTTGTTACGACTTCACCCCA_3'		
blaTEM	F-5´_ATGAGTATTCAACAT TTCCG_3´	956	23
	R-5´_CTGACAGTTACCAATGCTTA_3´		
bla VIM	F-5´_AAAGTTATGCCGCACTCACC_3´	865	24
	R-5´_TGCAACTTCATGTTATGCCG_3´		
bla IMP	F- 5´_ATGAGCAAGTTATCCTTATTC_3´	741	25
	R- 5´_GCTGCAACGACTTGTTAG_3´		
blaCTX-M-9	F -5´_GTGACAAAGAGAGTGCAACGG_3´	856	26
	R-5´_ATGATTCTCGCCGCTGAAGCC_3´		
blaOXA-51	F-5´_CGGAGAACGACTCCTCATTAAAAA_3´	431	27
	R-5´_TTTAGCTCGTCGTATTGGACTTGA_3´		
blaOXA-58	F-5´_AAGTATTGGGGCTTGTGCTG_3´	599	28
	R-5´_CCCCTCTGCGCTCTACATAC_3´		
Int1	F-5´_CAGTGGACATAAGCCTGTTC_3´	160	29
	R-5'_CCCGAGGCATAGACTGTA_3´		
Int2	F-5´_TTGCGAGTATCCATAACCTG_3´	288	29
	R-5´_TTACCTGCACTGGATTAAGC_3´		
CS	R-5´_GGCATCCAAGCAGCAAG_3´	Variable	29
	F-5´_AAGCAGACTTGACCTGA_3´		
hep35	R-5´_TGCGGGTYAARGATBTKGATTT_3´	491	30
hep36	F-5´_CARCACATGCGTRTARAT_3´

### Detection of integrase genes by PCR

The integrase class 1 *(intI)* and 2 *(intII*) genes were amplified as previously described ^15^ ^,^ ^30^. Amplification for each one was done in 50 µL volumes using 5-10 ng (genomic DNA) reaction buffer, 2 U of Taq polymerase (Bioline, London, United Kingdom), 200 µM each deoxynucleoside triphosphate, 2,0 mM MgCl_2_, 10 pmol of each primer. PCR conditions were as follows: a hot start at 94° C for 5 min; 94° C for 30 s by 35 cycles, 30 s at both 55° C; and a final step of 10 min at 72° C.

The reaction mixture to the degenerate primers hep35 and hep36 were equivalents to the previous except for the concentration of each primer (2.0 µM), and for the PCR extension steps (72^o^ C and 2 min).

### Statistical analysis

The data were analyzed using Stata version 11.0 (Stata Corp, College Station, Tex). Categorical variables were analyzed using χ^2^ test. All tests were two-tailed with *p* <0.05 considered significant. 

## Results

### Antibiotic susceptibility 

Antibiotyping tested by disk diffusion method showed multi-resistance to a large range of antibiotics. Most isolates of *Acinetobacter* spp. were resistant to trimethoprim /sulfamethoxazole, gentamicin, amikacin, tobramycin, ticarcillin/clavulanic acid, cefepime, ceftazidime, and imipenem. They were resistance rates of 100% while presenting variable susceptibility to ciprofloxacin (51/52, 98.1%), levofloxacin (45/52, 86.5%), ampicillin-sulbactam (49/52, 94.2%) and meropenem (50/52, 96.2%). The antimicrobial agents to which *Acinetobacter* spp strains were most susceptible were tigecycline and sulperozone. In contrast, 21.2% (11/52) and 28.8% (15/52) of them were resistant to tigecycline and sulperozone, respectively. In addition, all isolates were resistant to at least three classes of antibiotics (imipenem o meropenem, amikacin o tobramycin and extended-spectrum cephalosporins.), hence meeting the criteria for multidrug resistance ^17^. Remarkable, from the isolates circulated in July and September 2009, 10 isolates (19.3%) were considered Pan Drug Resistant (PDR) according to with Falagas *et al*. ^31^, demonstrating resistance to all antibiotics tested in this study. 

### A common antibiotype in unrelated co-circulating strains

Depending upon their susceptibilities to 14 different antimicrobial drugs, the 52 isolates of ACB complex were grouped into five (I to V) different antibiotypes. Most the isolates belonging to antibiotype I (50.0%), with fewer isolates belonging to antibiotype IV (19.3%), antibiotype II (17.3%) and antibiotype V, with only 1.9% of all the isolates. Interestingly, the isolates belonging to antibiotype I were susceptible to tigecycline and sulperozone, and most clinically significant isolates were found in nasal swabs (57.7%), followed by wound and catheter tips (23.1% and 15.4%, respectively) (*p* <0.05) (Table 2). Twenty-five isolates were collected between June and November of 2009. Only one isolate was detected in May 2010. 

**Table 2:** Antibiotypes of *A. baumannii-calcoaceticus complex* isolates by sample site, resistance and susceptibility profiles

Antibiotype	Isolates n (%)	Sample Site				Resistance profile			Susceptibility profile
		Nt n (%)	Bl n (%)	Ct n (%)	Wd n (%)	Ur n (%)	Us n (%)		
I	26 (50.0)	15 (57.7)	1 (3.8)	4 (15.4)	6 (23.1)	-	-	AMK, GEN, TOB, CIP, LVX, SXT, SAM, TIC/AC, FEP, CAZ, IMP, MEM	TIG, SUL
II	9 (17.3)	6 (66.7)	-	2 (22.2)	-	1 (11.1)	-	AMK, GEN, TOB, CIP, SXT, TIC/AC, FEP, CAZ, IMP	TIG, SUL/ LVX, SAM o MEM
III	6 (11.5)	1 (16.7)	2 (33.3)	-	1 (16.7)	2 (33.3)	-	AMK, GEN, TOB, CIP, LVX, SXT, SAM, TIC/AC, FEP, CAZ, IMP, MEM	TIG ó SUL
IV	10(19.3)*	2 (20.0)	-	-	5 (50.0)**	1 (10.0)	2 (20.0)	AMK, GEN, TOB, CIP, LVX, SXT, SAM, TIC/AC, FEP, CAZ, IMP, MEM, TIG, SUL	-
V	1 (1.9)	-	1 (100)	-	-	-	-	AMK, GEN, TOB, SXT, SAM, TIC/AC, FEP, CAZ, IMP, MEM	TIG, SUL, CIP, LVX
Total	52 (100)	24 (46.2)	4 (7.7)	6 (11.5)	12 (23.1)	4 (7.7)	2 (3.8)		

The antibiotype IV significantly grouped the PDR (19.3%) isolates (*p* <0.05) and was detected more frequently in wound (50%; OR = 5.000; *p*= 0.025). This antibiotype was first detected in March of 2009 and appeared again in July, August, and November of the same year, as well in April, May, and June of 2010. As shown in Table 2, 27 isolates of *A. baumannii* (37.1%) exhibited five distinct antibiotypes. Twelve of the isolates (54.5%) were classified as having antibiotype I, two (9.1%) isolates to antibiotype III, and four (18.2%) isolates to antibiotype and one (5%) isolate to antibiotype V. Four strains of *A. calcoaceticus* revealed two distinct antibiotypes, and three strains of 13TU showed three different antibiotypes.

### Identification of isolates of Acinetobacter spp

The genomic identification of 52 strains belonging to the ACB complex was performed using the ARDRA method and sequencing (Table 3, ^4^). 

**Table 3:** Distribution and molecular characterization of *A. baumannii-calcoaceticus complex* isolates.

Code	Sample Site	Antibiotype	*Bla* Genes						Integron
			OXA-51	TEM	CTXM-9	VIM-2	IMP-1	OXA-58	
5790341	Nasal trace	1	+						
5793548	Nasal trace	1	+	+				+	
21262	Urine	2	+						
5701177	Wound	1	+	+					+
1	Wound	4	+					+	+
5793546	Nasal trace	1	+			+	+		
5702062	Nasal trace	2	+						
5701373	Wound	1	+	+		+			+
5704225H	Wound	4	+					+	
5717479	Nasal trace	1	+						
5718164	Nasal trace	2	+					+	+
21311	Wound	1	+					+	
5728549	Wound	3	+			+		+	
21499	Nasal trace	1	+					+	
5730330	Catheter tips	1	+	+				+	
5748866	Blood	5	+						
5749428	Nasal trace	1	+			+		+	
5761126	Catheter tips	1	+					+	
5750598	U. secretion	4	+					+	
5723185	Nasal trace	1	+						+
2513	Urine	3	+			+	+		+
5759531	Nasal trace	4	+	+		+			+
209021(13TU)	Nasal trace	3						+	
5725011 (13TU)	Nasal trace	2			+			+	
5717971 (13TU)	Nasal trace	1				+		+	+
Ab3 *(A. calcoaceticus)*	Wound	1		+	+		+		
5701789 (*A. calcoaceticus)*	Nasal trace	4			+			+	
5701372 *(A. calcoaceticus)*	Nasal trace	1							
			85.7%	21.4%	10.7%	25.0%	10.7%	53.6%	28.6%

**Table 4:** *A. baumannii-calcoaceticus complex* profiles obtained by amplified rRNA gene restriction analysis (ARDRA)

No.	Genotype Sequence	ARDRA Profile	Isolates	Enzymes			
				*CfoI*	*AluI*	*MboI*	*MspI*
1	AB	AB	5704225H	1	1	1	1
2	AB	AB	Ab19606	1	1	1	1
3	AB	AB	2513	1	1	1	1
4	AB	AB	5748866	1	1	1	1
5	AB	AB	5790341	1	1	1	1
6	AB	AB	5730330	1	1	1	1
7	AB	AB	Ab21499	1	1	1	1
9	AB	AB	Ab21311	1	1	1	1
10	AB	AB	5728549	1	1	1	1
11	AB	AB	5717479	1	1	1	1
12	AB	AB	5793538	1	1	1	1
13	AB	AB	5761126	1	1	1	1
14	AB	AB	5702062	1	1	1	1
16	AB	AB	21262	1	1	1	1
17	AB	AB	Ab1	1	1	1	3
18	AB	AB	5701177	1	1	1	3
19	AB	AB	5759531	1	1	1	3
20	AB	AB	5750598	1	1	1	3
21	13TU	AB	209021	1	1	1	1
22	13TU	AB	5725011	1	1	1	1
23	13 TU	AB	5717971	1	1	1	3
24	UD	AB	5704581	1	1	1	1
25	*Calcoaceticus*	13TU	5701372	2	1	1	3
26	*Calcoaceticus*	13TU	5701789	2	1	1	1
27	UD	13TU	5748623	2	1	1	1
28	AB	3U	5793546	2	1	3	3
29	UD	3U	5738406	2	1	3	1
30	UD	UD	NAC51	2	2	4	1
							

The resistance profile were: *Calcoaceticus=A. calcoaceticus* (2 2 1 3); AB= *A. baumannii* (1 1 1 1 / 1 1 1 3); 3U= *Acinetobacter* 3U (2 1 3 3); *A. haemolyticus* (1 4 1 2); 13TU= *Acinetobacter* 13TU (2 1 1 1 / 2 1 1 3); UD= undefined

The results revealed that 23 (44.0%) isolates had the combined profile '1 1 1 2 3' or '1 1 1 2 1' for the several enzymes *CfoI, AluI, MboI, RsaI,* and *MspI*. According to the library of references profiles strains identified the organisms as species 2 (*A. baumannii*), three (5.8%) were *A. nosocomialis* (13TU), two were *A. calcoaceticus*, and two were *A. pitti* (genospecies 3).

For the NCBI-BLAST analysis, 27 strains were queried using sequences of a 1,500 bp fragment of the 16S rDNA. This identification of all clinical isolates to the species level showed that all amplicons had sequence concordance of 61 to 99% ^32^.

**Figure 1:**
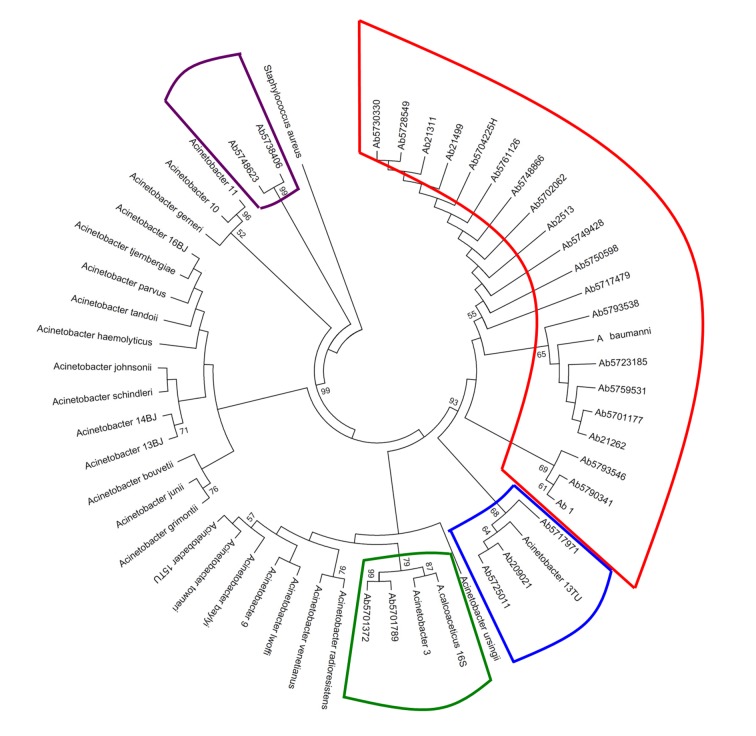
*Acinetobacter* genospecies phylogenetic tree by 16s rDNA sequencing analysis. In total, 27 clinical isolates were compared with Acinetobacter 16s rDNA16 sequences reported in the GenBank-NCBI. The phylogenetic tree was done using the MEGA software v.5 under Maximum Likelihood model with the Kimura-2-parameters, and Gamma distribution assuming invariable sites (K2+G+I). The robustness of phylogenetic tree was calculated by a bootstrap no parametric with 1000 replicates.

 In Figure 1, shown the Acinetobacter genospecies phylogenetic tree by 16s rDNA sequencing analysis. From all isolates, 74.1% (20/27) were clustering with *A. baumannii* while *A. nosocomialis* constituted 11.1% (3/27) of the total. However, we find ambiguity between the two methods when defining the genospecies *A. calcoaceticus, Acinetobacter 3U, Acinetobacter 13U* as shown in Table 4.

### Molecular characterization of ESBL genes

All ESBL-producing isolates in *ACB* complex were subjected to PCR experiments to detect the ESBL genes, including *blaTEM, blaSHV, blaCTX-M, blaOXA-51, blaOXA-58, blaVIM,* and *blaIMP*. Thirty-nine isolates (75.0%) carried several *bla* genes (up to two genes).

PCR amplification of class A beta-lactamase genes revealed that 17.3% (9/52) of the isolates carried *blaTEM-1* and five (9.6%) isolates contained bla_CTXM-9_, while bla_OXA-58_ was found significantly in all carbapenem-resistant *A. baumannii* strains (*p* <0.05). The *blaTEM-1* gene was identified in four isolates of *A. baumannii*, and one isolate of *A. calcoaceticus*. The *blaCTXM-9* gene was detected in two isolates of *A. calcoaceticus*, two isolates of *A. baumannii*, and one isolate of 13TU, the *blaOXA-58* gene was detected in once an isolate of *A. baumanni*, two isolates of *A. calcoaceticus*, and one isolate of 13TU. All *A. baumannii* isolates were positive for blaOXA-51-like genes. PCR did not detect the *blaOXA-23* and *blaSHV* genes.

Among MBL-producing isolates in *ACB* complex, *blaVIM-2* and *blaIMP-1* were found in 21.2%, and 7.7% of isolates, respectively. In 30.0% of the *A. baumannii* strains were detected the *blaVIM-2* gene, and all were obtained from nasal swabs.

### Detection of integrons by PCR

PCR detection of the intI1, intI2, and integrase genes demonstrated the presence of integrons in *Acinetobacter* species isolated. PCR performed the determination of the size of any inserted gene cassette within the genome. Overall, PCR of the integrase gene resulted in a frequency of integron-positive isolates of 38.5% (20/52) with various insert sizes within the *ACB* complex, in according to with previous studies that have reported a high frequency of multiresistant gram-negative isolates containing integrons ^(13-15,25,30)^.

Class 1 integrons were found in 23.1% (12/52) of the isolates of *ACB* complex. The length the amplicons of variable regions ranged between 0.75 to 2.5 kb; 0.75 kb (13.0%), 2.5 kb (5.0%) and (0.75 +2.5 kb) (7.0%). Further, PCR could be helpful to determine their gene cassette assortments. The most number of isolates with integrons belonging to antibiotype I and were found in nasal swabs and wounds. The *intI2* gene of class 2 integrons were detected in 17.3% (9/52) of the isolates. The presence of class 2 integrons, in six isolates, which also contained a class 1 integron was statistically significant (*p* <0.05).

Class 1 and 2 integrons were detected in two isolates belonging to antibiotype I and IV with *blaTEM-1* and *blaOXA* genes. Additionally, isolates belonging to antibiotype II and III also contained integrons. In contrast, no integrin-positive isolates were found in antibiotype V.

The integrons were found in 30.0% (7/23) isolates of *A. baumannii*. Class 1 integrons were detected in 26.1% (6/23) of the *A. baumannii* isolates, whereas only one *A. baumannii* strain contained a class 2 integron (Table 3). Integrons were detected in four (13.6%) *A. baumannii* isolates obtained from nasal swabs, two from (9.1%) from wounds, and one (4.5%) from urinary tracts, although the association was not statistically significant (*p* <0.05). Also, the integron-positive *A. baumannii* isolates contained *blaTEM-1*and *blaVIM-2* genes.

## Discussion

Nosocomial infections due to *A. baumannii* have been reported throughout the world ^5^ ^,^ ^33^. However, there is little information about the epidemiological behavior of the isolates circulating within the city of Cali. In this regard, the availability of 52 nosocomial isolates has offered the opportunity to assess the susceptibility profiles, the determinants of resistance to antibiotics and their mechanisms of resistance. The majority of the isolates of the *ACB* complex were more frequent in nasal tracking (46.2%), and of these, 63.6% corresponds to *A. baumannii* in patients admitted to the ICU. This finding is significant because the nasal colonization in patients older than 65 years with pre-existing lung disease or any debilitating diseases especially in the ICU has a higher risk to develop a Health care-associated infections (HAIs) ^34^ ^,^ ^35^.

On the other hand, it concerns that all the 52 isolates were multidrug resistant to antibiotics, and tigecycline only sulperazona maintained the antimicrobial activity against the majority of the isolates evaluated (80.0%). Also, we identified PDR isolates (19.3%), with a statistically significant value (*p* <0.05), a result similar to the one reported by some authors in Brazil in the same years of this study, where we found isolates with this feature in 11.0% ^31^. 

In Colombia, the resistance reported in *A. baumannii* has increased in recent years. Specifically, in Bogotá between the years 2001 and 2008, there were isolated strains sensitive to carbapenems, quinolones, and next-generation cephalosporins and aminoglycosides in 30% of the cases ^36^. Also, Pinzon *et al.* ^37^ for that same year, reported strains sensitive to carbapenems (35.8%); however, in 2012, the number of isolates with resistance to carbapenems had increased, still more than 90% ^38^.

In 2009, the PAHO reported in countries of Latin America percentages of carbapenem resistance above 70%, and similar results in the behavior of the resistance to aminoglycosides, trimethoprim-sulfamethoxazole in several countries of Central and South America ^39^. 

The reports of the SENTRY in countries of Latin America, Europe, and the United States indicate that Acinetobacter spp. present high rates of resistance even to carbapenem. A multicenter study conducted by Higgins *et al.* ^33^ in the year 2010 showed that 100% of the isolates of *A. baumannii* were resistant to imipenem, by the data obtained in this study for the same period. All of the isolated *ACB* complexes were multidrug resistant, including the isolates of *A. baumannii*, with percentages of carbapenem resistance more than 96%, which shows an increase in the resistance to antibiotics in the past few years. These findings are disturbing because the new antibacterial agents developed, such as doripenem, ceftobiprole, and ceftaroline, do not show activity against *A. baumannii* resistant to cephalosporins and carbapenems ^40^. Boo *et al*. ^41^, raise the possibility that a co-selection of isolates resistant to carbapenems occurs by the acquisition of carbapenemase OXA class D type. Our study showed that all isolates of *A. baumannii* presented the ability to hydrolyse broad-spectrum cephalosporins (ceftazidime and cefepime) and carbapenems (imipenem and meropenem). We detected the presence of beta-lactamase type OXA-51 and OXA-58, which would explain the resistance so marked to carbapenem, similarly reported by Gales *et al* ^42^.

This study demonstrated the poor capacity of *Acinetobacter* species identification by the Vitek-2 GNI system. It highlights the need to regard such results as preliminary data. Accurate identification using molecular methods is not only important in the investigation of outbreaks caused by Acinetobacter species, but also is relevant in epidemiological studies such as this report. Our results are like the reports published in other geographic regions ^43^ and coincide with the reports of Karageorgopoulos *et al*. ^44^, who found sensitivity to tigecycline lower than 90% of the isolates of *Acinetobacter spp*. They make it a potentially useful treatment option against highly resistant bacteria; however, few of these bacteria also present resistance to tigecycline. 

Within multiresistant isolates of the *ACB* complex and those of *A. baumannii, bla* (*TEM-1, CTX-9, OXA-58, IMP-1 and VIM-2*) was detected. Although the presence of the beta-lactamase TEM-1 is reported as one of the leading causes of resistance to beta-lactamic antibiotics in the *A. baumannii* isolates, in this study, only 22.7 % of the isolates were carriers of the gene *blaTEM-1*. In recent years the trend has changed; the increase in the resistance to carbapenems is mainly due to the presence of metallo beta-lactamases and class D type carbapenemase OXA, whose overexpression is regulated by the presence of upstream insertion elements, such as *ISAba1* ^11^.

In this study, 38.5% of the isolates of *ACB* complex and 50.0% of *A. baumannii* presented beta-lactamases type OXA-58. Despite that, some reports have suggested that genes of OXA type beta-lactamases are transported in integrons ^37^ ^,^ ^38^, our results showed that 82% of the isolates of the ACB complex and *A. baumannii* that contained these genes type did not show related to integrons. These results are consistent with those reported by Poirel et al. ^45^, who said that these genes are not usually found in the form of gene cassettes and, according to the same author, these genes are carried on plasmids or are associated with a process of homologous recombination. 

The presence of integrons (class 1 and 2) in 40% of the isolates was statistically significant; both classes of integrons have been described between the members of the genus Acinetobacter isolated in both clinical and environmental settings ^14^. It is reported that the epidemic strains of *A. baumannii* tend to contain a greater number of integrons that are non-epidemic, and affirms that the use of antibiotics has a high impact on the development of the diversity and maintenance of these strains in the ICU ^46^. 

The class 1 integron presents multiple cassettes, which confers resistance towards several antibiotics, as a distinctive phenotypic stamp on the isolates of *A. baumanni* ^13^ ^-^ ^15^ ^,^ ^30^, which would explain the multidrug resistance detected in isolates of the *ACB* complex and *A. baumannii*. The integration of these elements in the bacterial chromosome can affect the expression of genes such as *blaOXA-51,* which encodes an Amp c, b, and the chromosomal carbapenemase. In this study, all 22 isolates of *A. baumannii* showed resistance to cephalosporins and carbapenems; however, only 25% of them detected integrons within the genome. Of the *ACB* complex, 32 isolates were not integrons, by which the multidrug resistance to antibiotics in these isolates must be related to other mechanisms of resistance which may be plasmid-mediated or through the reduction of the cell membrane or wall permeability ^47^. The largest numbers of integrons were detected in isolates obtained from sample tracking and nasal surgical wounds. These results do not agree with those obtained by Koleman *et al*. ^29^, whereas that the largest number of integrons was detected in isolates from blood and secretion; this discrepancy is probably due to the greater number of isolates of this study that were obtained from nasal colonization and absence of infections. 
