# Supplementary material for: Characterization of multidrug-resistant Acinetobacter ssp. strains isolated from medical intensive care units in Cali - Colombia
Source: Colomb Med (Cali). 2017 Dec 30;48(4):183–90. doi: 10.25100/cm.v48i4.2858 (PMC5896725; doi:10.25100/cm.v48i4.2858)
Supplement: Related texts

# Research article: Caracterización de cepas de ***Acinetobacter*** ***spp*** . resistentes a múltiples fármacos aisladas de unidades de cuidados intensivos en Cali - Colombia

## Introducción

Se ha reportado que *Acinetobacter* spp. está involucrada en infecciones adquiridas en el hospital con una frecuencia creciente ^1^. El uso extensivo de la quimioterapia antimicrobiana en entornos clínicos ha contribuido a la aparición y diseminación de infecciones nosocomiales por *Acinetobacter* spp ^2^. Las especies pertenecientes al complejo *A. calcoaceticus- A. baumannii* (complejo ACB) son principalmente cepas de *Acinetobacter* resistentes a antibióticos ^3^. Este complejo ha sido implicado como la causa de un amplio espectro de enfermedades infecciosas como neumonía, meningitis, bacteriemia, infecciones del tracto urinario e infecciones relacionadas con los dispositivos especialmente en unidades de cuidados intensivos (UCI), y asociadas a altas tasas de mortalidad ^4^. Debido al fenotipo resistente a múltiples fármacos (MDR, siglas en inglés) del organismo, estas infecciones son difíciles de tratar, lo que incluye resistencia a betalactámicos, aminoglucósidos, fluoroquinolonas y carbapenémicos ^5^^,^^6^. En los últimos años se ha producido un brote nosocomial significativo del complejo complejo MDR ACB en Colombia ^7^.

La resistencia a los betalactámicos, incluidos los carbapenémicos en el complejo ACB, se debe principalmente a la producción de betalactamasas de espectro extendido (ESBLs), pero también a otros mecanismos, como alteraciones en proteínas de la membrana externa y proteínas de unión a penicilina y aumento de la actividad de las bombas de explusión ^8^. Tanto las ESBLs, carbapenemasas, como las metalo-beta-lactamasas (MBL) o las oxacilinasas, representan la mayor preocupación debido a la posibilidad de una diseminación rápida ^9^. Mientras que las MBL encontradas son de tipos IMP y VIM en *A. baumannii*^5^, las oxacilinasas presentan cuatro subgrupos principales de OXA que son cromosómicamente localizados como similar a OXA-51 intrínseco; similar a OXA-23 adquirido; similar a OXA-40; y similar a OXA-58 ^9^^-^^12^.

La mayoría de los genes que codifican estos ESBLs se encuentran en plásmidos, o en forma de un casete genético en un integrón. Los integrones son elementos genéticos que poseen un sitio de recombinación particular, conocido como *attl*1, en el cual los genes de resistencia pueden insertarse mediante recombinación sitio específica en forma de casetes de genes ^13^. Se han descrito diferentes clases de integrones, y las clases 1, 2 y 3 se han asociado con resistencia a antibióticos ^13^^,^^14^. La diseminación de estos integrones de resistencia a antibióticos, que no pueden promover su movilización, está principalmente relacionada con transposones y plásmidos. Se han publicado diferentes informes que identifican a los integrones como responsables de la presencia y adquisición de resistencia a antibióticos en *A. baumannii*^13^^,^^14^. Hay datos limitados sobre la epidemiología global de *A. baumannii* en nuestra región. Sin embargo, la distribución del integrón de clase 2 es muy frecuente en aislamientos clínicos de *A. baumannii* de Argentina, Chile y Brasil ^14^^,^^15^.

Los objetivos del presente estudio fueron analizar las características fenotípicas y moleculares del complejo de ACB y la resistencia a antibióticos en aislados clínicos en un hospital colombiano de atención terciaria. Además de determinar los perfiles de resistencia antimicrobiana de las cepas aisladas. También se comprobó la presencia de MBL o ESBL en *Acinetobacter spp*.

Para probar la hipótesis de que la mayoría de los genes que codifican MBL o ESBL se encuentran en los casetes génicos de los integrones, se examinaron por PCR, en aislados representativos, la presencia de los integrones de clase 1 y clase 2 y los genes asociados a resistencia antimicrobiana. El conocimiento sobre la epidemiología y los mecanismos moleculares de la resistencia a los antimicrobianos en este importante patógeno es esencial para implementar estrategias de intervención.

## Materiales y Métodos

Se llevó a cabo un estudio descriptivo respaldado por el comité de ética del hospital.

### Muestras clínicas de cepas bacterianas

El estudio incluyó 52 cepas de *Acinetobacter* spp obtenidas de muestras clínicas, previamente identificadas por la tarjeta Vitek GNI (bioMeriex Vitek Inc., Hazelwood, MO), que crecieron en agar MacConkey estándar a 37 °C durante 48 h, entre marzo de 2009 y julio de 2010 de una unidad de cuidados intensivos médicos en el Hospital Universitario Rafael Uribe Uribe de Cali - Colombia. Las muestras clínicas se obtuvieron de: Rastreo nasal, 24 (46.2%); heridas, 12 (26.1%); punta del catéter, 6 (11.6%); el tracto urinario, 6 (11.6%); y sangre, 4 (7.7%). Todas las muestras se almacenaron a -80 °C en caldo nutritivo con 15% de glicerol. De las 52 cepas aisladas de *Acinetobacter* spp, se obtuvieron 24 (46.2%).

### Prueba de sensibilidad antimicrobiana

La susceptibilidad a varias clases de antibióticos se determinó mediante el método de difusión de disco en un agar Mueller-Hinton (MH) (Difco Laboratories, Detroit, MI, EE. UU.), según las directrices del Instituto Clínico y de Laboratorio Estándar (CLSI) ^16^. Ambas cepas, *Acinetobacter baumannii* (ATCC(r) 19606 (tm)) y *Escherichia coli* (ATCC(r) 25922 (tm)) se usaron como cepas de control de calidad. Los discos de antibióticos (Oxoid(r)) probados fueron los siguientes: trimetroprima/sulfametoxazol (SXT, 23.75 μg/1.25 μg); ticarcilina/clavulanato (TIM, 75 μg/10 μg); gentamicina (GEN, 10 μg); tobramicina (TOB, 10 μg); ciprofloxacina (CIP, 5 μg); ceftazidima (CAZ, 30 μg); cefepime (FEP, 30 μg); aztreonam (ATM, 30 μg); imipenem (IMP, 10 μg); meropenem (MEM, 10 μg); ampicilina/sulbactam (SAM, 10 μg/10 μg); amikacina (AMK, 10 μg); cefoprazona/sulbactam (sulperazona, SUL, 75 μg/30 μg); y tigeciclina (TIG, 15 μg). De acuerdo con Manchanda *et al.*^17^ se definió *Acinetobacter* spp*.* MDR si los aislados son resistentes a tres antibióticos diferentes a la ceftazidima, como ciprofloxacina, gentamicina e imipenem.

### Identificación de aislados de Acinetobacter spp.: Análisis de Restricción de ADN Ribosomal Amplificado (ARDRA)

La especie genómica de *Acinetobacter* spp. fue determinada por ARDRA, de acuerdo con Vaneechoutte *et al*^18^. En resumen, el ADN de un cultivo de una noche en caldo LB a 37°C fue extraído utilizando el kit "Easy-DNA^TM^" (Invitrogen, life technologies (r)) de acuerdo con las instrucciones del fabricante. Después de la electroforesis, el gel se tiñó con bromuro de etidio (1.5 μg/mL) y se analizó en un FOTO/Analyst(r) Investigator/FX Systems (FOTODYNE Incorporated). Para cada muestra, la reacción de PCR se realizó para la amplificación del gen 16s ADNr utilizando el siguiente par de cebadores: Acf5'-TGG CTC AGA TTG AAC GCT GGC GGC-3'y Acr5'-TAC CTT GTT ACG ACT TCA CCC CA-3'. Los productos de PCR se digirieron con las siguientes enzimas: CfoI; Alu; Mbo; y MspI (Fermentas (tm)) y separados por electroforesis en gel de poliacrilamida (PAGE 8.0%).

Los últimos perfiles de restricción se compararon con la base de datos de la biblioteca de cepas (http://users.ugent.be/~mvaneech/ARDRA/Acinetobacter.html) para identificar la especie de acuerdo con Dijkshoorn *et al* ^19^. La cepa de A. baumannii (ATCC(r) 19606 (tm)) se usó como control.

### Identificación de aislados clínicos de Acinetobacter spp. mediante PCR-secuenciación del gen 16s ADNr

Se purificaron 27 productos de PCR para el gen 16s ADNr, usando el kit "High Pure PCR Purification Kit Version 20, de acuerdo con las instrucciones de la compañía (Roche Applied Science^®)^ y luego, cada producto de PCR purificado se secuenció directamente mediante el método dideoxi de Sanger usando un Secuenciador ABI 3730XL. Las secuencias de 16S ADNr se alinearon con 27 secuencias de referencia de *Acinetobacter* albergadas en la base de datos GenBank-NCBI.

El árbol filogenético se realizó utilizando el software MEGA v.5 bajo el modelo de Máxima Verosimilitud con los parámetros Kimura-2, y la distribución Gamma asumiendo sitios invariables (K2 + G + I). La robustez del árbol filogenético se calculó mediante un bootstrap no paramétrico con 1,000 réplicas ^20^^,^^21^. Se utilizó una secuencia de *Staphylococcus aureus* como grupo externo.

### Detección de genes de ESBL por PCR

Genes de ESBL, tales como *bla*TEM; *bla*SHV; *bla*OXA; *bla*CTX-M; *bla*IMP; y *bla*VIM, se detectaron por PCR usando los cebadores enumerados en la Tabla 1 ^22^^-^^30^. El volumen final de la reacción de PCR fue de 50 μL con 5-10 ng (ADN genómico) buffer de reacción, 1 U de Taq polimerasa (Bioline, Londres, Reino Unido), 200 μM para cada desoxinucleósido trifosfato, 1.5 o 2.5 mM MgCl_2_, 10 pmol de cada primer. La temperatura del termociclador fue de 94° C durante 5 min; 94° C durante 30 s por 35 ciclos. Para *bla*TEM, 52° C durante 45 s; *bla*OXA-51 y *bla*OXA-58, 62° C durante 1 minuto; *bla*CXT-M, 51° C durante 45 segundos; *bla*VIM y *bla*IMP 51° C durante 1 min; y 72° C durante 60 s. Una etapa final de 10 min a 72° C.

**Tabla 1:** Cebadores usados en el estudio

Cebador	Secuencia de oligonucleotidos	Tamaño (bp)	Referencia
rDNA16S AC	F-5'_TGGCTCAGATTGAACGCTGGCGGC_3'	1,500	18
	R-5'_TACCTTGTTACGACTTCACCCCA_3'		
blaTEM	F-5´_ATGAGTATTCAACAT TTCCG_3´	956	23
	R-5´_CTGACAGTTACCAATGCTTA_3´		
bla VIM	F-5´_AAAGTTATGCCGCACTCACC_3´	865	24
	R-5´_TGCAACTTCATGTTATGCCG_3´		
bla IMP	F- 5´_ATGAGCAAGTTATCCTTATTC_3´	741	25
	R- 5´_GCTGCAACGACTTGTTAG_3´		
blaCTX-M-9	F -5´_GTGACAAAGAGAGTGCAACGG_3´	856	26
	R-5´_ATGATTCTCGCCGCTGAAGCC_3´		
blaOXA-51	F-5´_CGGAGAACGACTCCTCATTAAAAA_3´	431	27
	R-5´_TTTAGCTCGTCGTATTGGACTTGA_3´		
blaOXA-58	F-5´_AAGTATTGGGGCTTGTGCTG_3´	599	28
	R-5´_CCCCTCTGCGCTCTACATAC_3´		
Int1	F-5´_CAGTGGACATAAGCCTGTTC_3´	160	29
	R-5'_CCCGAGGCATAGACTGTA_3´		
Int2	F-5´_TTGCGAGTATCCATAACCTG_3´	288	29
	R-5´_TTACCTGCACTGGATTAAGC_3´		
CS	R-5´_GGCATCCAAGCAGCAAG_3´	Variable	29
	F-5´_AAGCAGACTTGACCTGA_3´		
hep35	R-5´_TGCGGGTYAARGATBTKGATTT_3´	491	30
hep36	F-5´_CARCACATGCGTRTARAT_3´

### Detección de genes de integrasa por PCR

Los genes de la integrasa clase 1 (intI) y 2 (intII) se amplificaron como se describió previamente ^15^^,^^30^. La amplificación para cada uno se realizó en volúmenes de 50 μL usando 5-10 ng (ADN genómico) de buffer de reacción, 2 U de polimerasa Taq (Bioline, Londres, Reino Unido), 200 μM para cada desoxinucleósido trifosfato, 2,0 mM de MgCl_2_, 10 pmol de cada cebador. Las condiciones de PCR fueron las siguientes: un inicio con choque de calor a 94 °C durante 5 min; 35 ciclos de 30 s a 94 °C, 30 s a 55 °C; y un paso final de 10 min a 72 °C.

La mezcla de reacción para los cebadores degenerados hep35 y hep36 eran equivalentes a los anteriores excepto por la concentración de cada cebador (2.0 μM) y para los pasos de extensión de la PCR (72º C y 2 min).

### Análisis estadístico

Los datos se analizaron usando Stata versión 11.0 (Stata Corp, College Station, Tex). Las variables categóricas se analizaron mediante la prueba χ^2^. Todas las pruebas fueron de dos colas con *p* <0.05 considerado significativo.

## Resultados

### Susceptibilidad a antibióticos

Los antibióticos probados por el método de difusión en disco mostraron resistencia múltiple a una amplia gama de antibióticos. La mayoría de los aislamientos de *Acinetobacter* spp*.* fueron resistentes a trimetoprima / sulfametoxazol, gentamicina, amikacina, tobramicina, ticarcilina/ácido clavulánico, cefepime, ceftazidima e imipenem con tasas de resistencia del 100%; mientras presentaban susceptibilidad variable a ciprofloxacina (51/52, 98.1%), levofloxacina (45/52, 86.5%), ampicilina-sulbactam (49/52, 94.2%) y meropenem (50/52, 96.2%). Los agentes antimicrobianos a los que las cepas de *Acinetobacter* spp fueron más susceptibles fueron la tigeciclina y la sulperozona. Por el contrario, el 21.2% (11/52) y el 28.8% (15/52) de ellos fueron resistentes a tigeciclina y sulperozona, respectivamente. Además, todos los aislamientos fueron resistentes a al menos tres clases de antibióticos (imipenem o meropenem, amikacina o tobramicina y cefalosporinas de espectro extendido), cumpliendo así los criterios de resistencia a múltiples fármacos ^17^. Es de resaltar, que de los aislamientos distribuidos en julio y septiembre de 2009, 10 aislamientos (19.3%) se consideraron Pan resistente a las drogas (PDR) de acuerdo con Falagas *et al.*^31^, demostrando resistencia a todos los antibióticos probados en este estudio.

### Un antibiótico común en cepas no relacionadas de co-circulación

Dependiendo de sus susceptibilidades a 14 diferentes fármacos antimicrobianos, los 52 aislamientos del complejo ACB se agruparon en cinco (I a V) antibiotipos diferentes. La mayoría de los aislamientos pertenecientes al antibiotipo I (50.0%), con menos aislados pertenecientes al antibiotipo IV (19.3%), antibiotipo II (17.3%) y antibiotipo V, con solo 1.9% de todos los aislados. Curiosamente, los aislados pertenecientes al antibiótipo I eran susceptibles a tigeciclina y sulperozona, y la mayoría de los aislados clínicamente significativos se encontraban en rastreo nasal (57.7%), seguidos de heridas y puntas de catéter (23.1% y 15.4%, respectivamente) (*p* <0.05) (Tabla 2). Del antibiotipo I, veinticinco aislados fueron recolectados entre junio y noviembre de 2009 y solo se detectó un aislado en mayo de 2010.

**Tabla 2:** Antibiotipos del complejo *A. baumannii-calcoaceticus* aislados del sitio de muestreo, perfiles de resistencia y susceptibilidad

Antibiotipo	Aislados n (%)	Sitio de muestra				Perfil de resistancIA			Perfil de susceptibilidad
		MN n (%)	San n (%)	PC N (%)	He n (%)	Or n (%)	SU n (%)		
I	26 (50.0)	15 (57.7)	1 (3.8)	4 (15.4)	6 (23.1)	-	-	AMK, GEN, TOB, CIP, LVX, SXT, SAM, TIC/AC, FEP, CAZ, IMP, MEM	TIG, SUL
II	9 (17.3)	6 (66.7)	-	2 (22.2)	-	1 (11.1)	-	AMK, GEN, TOB, CIP, SXT, TIC/AC, FEP, CAZ, IMP	TIG, SUL/ LVX, SAM o MEM
III	6 (11.5)	1 (16.7)	2 (33.3)	-	1 (16.7)	2 (33.3)	-	AMK, GEN, TOB, CIP, LVX, SXT, SAM, TIC/AC, FEP, CAZ, IMP, MEM	TIG ó SUL
IV	10(19.3)*	2 (20.0)	-	-	5 (50.0)**	1 (10.0)	2 (20.0)	AMK, GEN, TOB, CIP, LVX, SXT, SAM, TIC/AC, FEP, CAZ, IMP, MEM, TIG, SUL	-
V	1 (1.9)	-	1 (100)	-	-	-	-	AMK, GEN, TOB, SXT, SAM, TIC/AC, FEP, CAZ, IMP, MEM	TIG, SUL, CIP, LVX
Total	52 (100)	24 (46.2)	4 (7.7)	6 (11.5)	12 (23.1)	4 (7.7)	2 (3.8)		

El antibiotipo IV agrupó significativamente los aislados de PDR (19.3%) (*p* <0.05) y se detectó con mayor frecuencia en heridas (50%; OR = 5.000; *p* = 0.025). Este antibiotipo se detectó por primera vez en marzo de 2009 y apareció nuevamente en julio, agosto y noviembre del mismo año, así como en abril, mayo y junio de 2010. Como se muestra en la Tabla 2, 27 aislamientos de *A. baumannii* (37.1%) exhibieron cinco antibiotipos distintos. Doce de los aislados (54.5%) se clasificaron como antibiotipo I, dos (9.1%) aislados para antibiotipo III, cuatro (18.2%) aislamientos para antibiotipo IV y un (5.0%) aislado para antibiotipo V. Cuatro cepas de *A. calcoaceticus* revelaron dos antibiotipos distintos, y tres cepas de 13TU mostraron tres antibiotipos diferentes.

### Identificación de aislados de Acinetobacter spp

La identificación genómica de 52 cepas pertenecientes al complejo ACB se realizó mediante el método ARDRA y secuenciación (Tabla 3 y ^4^). Los resultados revelaron que 23 (44.2%) aislamientos tenían el perfil combinado "1 1 1 2 3" o "1 1 1 2 1" para las diversas enzimas CfoI, AluI, MboI, RsaI y MspI. De acuerdo con la biblioteca de referencias, las cepas de perfiles identificaron los organismos como especies 2 (*A. baumannii*), tres (5.8%) fueron *A. nosocomialis* (13TU), dos fueron *A. calcoaceticus* y dos fueron *A. pitti* (genospecies 3).

Para el análisis NCBI-BLAST, se consultaron 27 cepas utilizando secuencias de un fragmento de 1,500 pb del ADNr 16s. Esta identificación de todos los aislados clínicos a nivel de especie mostró que todos los amplicones tenían una concordancia de secuencia de 61 a 99% ^32^.

**Tabla 3:** Distribución y caracterización molecular de aislados del complejo *A. baumannii-calcoaceticus*.

Código	Sitio de muestreo	Antibiotipo	Genes *Bla*						Integron
			OXA-51	TEM	CTXM-9	VIM-2	IMP-1	OXA-58	
5790341	Muestra nasal	1	+						
5793548	Muestra nasal	1	+	+				+	
21262	Orina	2	+						
5701177	Herida	1	+	+					+
1	Herida	4	+					+	+
5793546	Muestra nasal	1	+			+	+		
5702062	Muestra nasal	2	+						
5701373	Herida	1	+	+		+			+
5704225H	Herida	4	+					+	
5717479	Muestra nasal	1	+						
5718164	Muestra nasal	2	+					+	+
21311	Herida	1	+					+	
5728549	Herida	3	+			+		+	
21499	Muestra nasal	1	+					+	
5730330	Punta cateter	1	+	+				+	
5748866	Sangre	5	+						
5749428	Muestra nasal	1	+			+		+	
5761126	Punta Cateter	1	+					+	
5750598	Secreción uretra	4	+					+	
5723185	Muestra nasal	1	+						+
2513	Orina	3	+			+	+		+
5759531	Muestra nasal	4	+	+		+			+
209021(13TU)	Muestra nasal	3						+	
5725011 (13TU)	Muestra nasal	2			+			+	
5717971 (13TU)	Muestra nasal	1				+		+	+
Ab3 *(A. calcoaceticus)*	Herida	1		+	+		+		
5701789 (*A. calcoaceticus)*	Muestra nasal	4			+			+	
5701372 *(A. calcoaceticus)*	Muestra nasal	1							
			85.7%	21.4%	10.7%	25.0%	10.7%	53.6%	28.6%

**Tabla 4:** Perfiles obtenidos por analisis de restriccion de genes amplificando rRNA del complejo *A. baumannii-calcoaceticus*

No.	Secuencia genotipo	Perfil ARDRA	Aislados	Enzimas			
				*CfoI*	*AluI*	*MboI*	*MspI*
1	AB	AB	5704225H	1	1	1	1
2	AB	AB	Ab19606	1	1	1	1
3	AB	AB	2513	1	1	1	1
4	AB	AB	5748866	1	1	1	1
5	AB	AB	5790341	1	1	1	1
6	AB	AB	5730330	1	1	1	1
7	AB	AB	Ab21499	1	1	1	1
9	AB	AB	Ab21311	1	1	1	1
10	AB	AB	5728549	1	1	1	1
11	AB	AB	5717479	1	1	1	1
12	AB	AB	5793538	1	1	1	1
13	AB	AB	5761126	1	1	1	1
14	AB	AB	5702062	1	1	1	1
16	AB	AB	21262	1	1	1	1
17	AB	AB	Ab1	1	1	1	3
18	AB	AB	5701177	1	1	1	3
19	AB	AB	5759531	1	1	1	3
20	AB	AB	5750598	1	1	1	3
21	13TU	AB	209021	1	1	1	1
22	13TU	AB	5725011	1	1	1	1
23	13 TU	AB	5717971	1	1	1	3
24	UD	AB	5704581	1	1	1	1
25	*Calcoaceticus*	13TU	5701372	2	1	1	3
26	*Calcoaceticus*	13TU	5701789	2	1	1	1
27	UD	13TU	5748623	2	1	1	1
28	AB	3U	5793546	2	1	3	3
29	UD	3U	5738406	2	1	3	1
30	UD	UD	NAC51	2	2	4	1
							

Los perfiles de resistencia fueron: *Calcoaceticus=A. calcoaceticus* (2 2 1 3); AB= *A. baumannii* (1 1 1 1 / 1 1 1 3); 3U= *Acinetobacter* 3U (2 1 3 3); *A. haemolyticus* (1 4 1 2); 13TU= *Acinetobacter* 13TU (2 1 1 1 / 2 1 1 3); UD= indefinido

En la Figura 1, se muestra el árbol filogenético de las genoespecies de *Acinetobacter* mediante el análisis de secuenciación del ADNr 16s. De todos los aislamientos, el 74.1% (20/27) se agruparon con *A. baumannii* mientras que *A. nosocomialis* constituyó el 11.1% (3/27) del total. Los métodos moleculares (ARDRA y análisis de secuencia) aplicados en este estudio nos permitieron discriminar dentro del complejo ACB cada una de las especies que lo componen. Sin embargo, encontramos ambigüedad entre los dos métodos al definir las genoespecies *A. calcoaceticus, Acinetobacter* 3U, *Acinetobacter* 13U como se muestra en la Tabla 4.

**Figura 1:** Árbol filogenético de *Acinetobacter* genospecies por análisis de secuenciación de rDNA 16s. En total, 27 aislados clínicos se compararon con las secuencias de *Acinetobacter* 16s rDNA16 informadas en el GenBank-NCBI. El árbol filogenético se realizó utilizando el software MEGA v.5 bajo el modelo de Máxima Verosimilitud con los parámetros Kimura-2, y la distribución Gamma asumiendo sitios invariables (K2 + G + I). La robustez del árbol filogenético se calculó mediante un bootstrap no paramétrico con 1,000 réplicas.

### Caracterización molecular de genes de ESBL

Todos los aislados productores de ESBL en el complejo ACB se sometieron a experimentos de PCR para detectar los genes ESBL, que incluyen *bla*TEM, *bla*SHV, *bla*CTX-M, *bla*OXA-51, *bla*OXA-58, *bla*VIM y *bla*IMP. Treinta y nueve aislamientos (75.0%) portaban varios genes bla (hasta dos genes).

La amplificación por PCR de los genes de beta-lactamasa de clase A reveló que el 17.3% (9/52) de los aislados contenían *bla*TEM-1 y cinco (9.6%) aislados contenían *bla*CTXM-9, mientras que *bla*OXA-58 se encontró significativamente en todas cepas de *A. baumannii* resistentes a carbapenem (*p* <0.05). El gen *bla*TEM-1 se identificó en cuatro aislados de *A. baumannii* y un aislado de *A. calcoaceticus*. El gen *bla*CTXM-9 se detectó en dos aislados de *A. calcoaceticus* y un aislado de 13TU, el gen *bla*OXA-58 se detectó en un aislado de *A. baumanni*, dos aislados de *A. calcoaceticus* y un aislado de 13TU. Todos los aislamientos de *A. baumannii* fueron positivos para genes similares a *bla*OXA-51. La PCR no detectó los genes *bla*OXA-23 y *bla*SHV.

Entre los aislamientos productores de MBL en el complejo ACB, *bla*VIM-2 y *bla*IMP-1 se encontraron en 21.2% y 7.7% de los aislamientos, respectivamente. En 30.0% de las cepas de *A. baumannii* se detectó el gen *bla*VIM-2, y todas se obtuvieron de rastreos nasales.

### Detección de integrones por PCR

La detección por PCR de los genes intl1, intl2 e integrasa demostró la presencia de integrones en las especies de *Acinetobacter* aisladas. La PCR realizó la determinación del tamaño de cualquier casete de gen insertado dentro del genoma. En general, la PCR del gen de la integrasa dio como resultado una frecuencia de aislamientos positivos para el integrón del 38.5% (20/52) con diversos tamaños de insertos dentro del complejo ACB, de acuerdo con estudios previos que informaron una alta frecuencia de gramnegativos multirresistentes aislados que contienen integrones ^13^^-^^15^^,^^25^^,^^30^.

Los integrones de clase 1 se encontraron en el 23.1% (12/52) de los aislamientos del complejo ACB. La longitud de los amplicones de las regiones variables oscilaba entre 0.75 y 2.5 kb; 0.75 kb (13.0%), 2.5 kb (5.0%) y (0.75 + 2.5 kb) (7.0%). Adicionalmente, la PCR podría ser útil para determinar sus surtidos de casete genético. La mayoría de los aislamientos con integrones pertenecientes al antibiotipo I se encontraron en rastreos nasales y heridas. El gen intI2 de los integrones de clase 2 se detectó en el 17.3% (9/52) de los aislados. La presencia de integrones de clase 2, en seis aislamientos, que también contenían un integrón de clase 1 fue estadísticamente significativa (*p* <0.05%).

Se detectaron integrones de clase 1 y 2 en dos aislados pertenecientes a los antibiotipos I y IV con los genes *bla*TEM-1 y *bla*OXA. Además, los aislados pertenecientes a los antibiotipo II y III también contenían integrones. Por el contrario, no se encontraron cepas con integrasa positiva en el antibiotipo V.

Los integrones se encontraron en 30.4% (7/23) aislados de *A. baumannii*. Se detectaron integrones de clase 1 en 26.1% (6/23) de los aislamientos de *A. baumannii*, mientras que solo una cepa de *A. baumannii* contenía un integrón de clase 2 (Tabla 3). Los integrones se detectaron en cuatro aislamientos de *A. baumannii* (13.6%) obtenidos de rastreos nasales, dos (9.1%) de las heridas y uno (4.5%) de las vías urinarias, aunque la asociación no fue estadísticamente significativa (*p* <0.05). Además, los aislamientos de *A. baumannii* positivos para el integrón contenían los genes *bla*TEM-1 y *bla*VIM-2.

## Discusión

Se han notificado infecciones nosocomiales por *A. baumannii* en todo el mundo ^5^^,^^33^. Sin embargo, hay poca información sobre el comportamiento epidemiológico de los aislados que circulan en la ciudad de Cali. En este sentido, la disponibilidad de 52 aislados nosocomiales ha ofrecido la oportunidad de evaluar los perfiles de susceptibilidad, los determinantes de la resistencia a los antibióticos y sus mecanismos de resistencia. La mayoría de los aislamientos del complejo ACB fueron más frecuentes en el rastreo nasal (46.2%) y de éstos, el 63.6% corresponde a *A. baumannii* en pacientes ingresados ​​en la UCI. Este hallazgo es significativo porque la colonización nasal en pacientes mayores de 65 años con enfermedad pulmonar preexistente o cualquier enfermedad debilitante, especialmente en la UCI, tiene un mayor riesgo de desarrollar infecciones asociadas a la atención médica (IAH) ^34^^,^^35^.

Por otro lado, preocupa que los 52 aislamientos fueran multi-resistentes a los antibióticos, y la tigeciclina y sulperazona fueran las únicas que mantuvieron la actividad antimicrobiana contra la mayoría de los aislados evaluados (80.0%). Además, se identificaron aislamientos de PDR (19.3%), con un valor estadísticamente significativo (p≤0.05), un resultado similar al reportado por algunos autores en Brasil en los mismos años de este estudio, donde se encontraron aislamientos con esta característica en 11% ^31^.

En Colombia, la resistencia informada en *A. baumannii* ha aumentado en los últimos años. Específicamente, en Bogotá entre los años 2001 y 2008, hubo cepas aisladas sensibles a carbapenémicos, quinolonas, y a cefalosporinas de última generación y aminoglucósidos en el 30% de los casos ^36^. Además, Pinzon *et al.* ^(^^37^, para ese mismo año, reportó cepas sensibles a carbapenémicos (35.8%); sin embargo, en 2012, el número de aislamientos con resistencia a carbapenémicos aumentó, en más del 90% ^38^.

En 2009, la PAHO informó en los países de América Latina porcentajes de resistencia a carbapenem por encima del 70%, y resultados similares en el comportamiento de la resistencia a los aminoglucósidos, trimetoprim-sulfametoxazol en varios países de América Central y del Sur ^39^.

Los informes de SENTRY en países de América Latina, Europa y los Estados Unidos indican que *Acinetobacter spp*. presenta altas tasas de resistencia incluso al carbapenem. Un estudio multicéntrico realizado por Higgins *et al.* ^(^^33^, en el año 2010 mostró que el 100% de los aislamientos de *A. baumannii* eran resistentes a imipenem, lo que coincide con los datos obtenidos en este estudio durante el mismo período. Todos los complejos ACB aislados fueron resistentes a múltiples fármacos, incluidos los aislados de *A. baumannii*, con porcentajes de resistencia a carbapenem en más del 96%, lo que muestra un aumento en la resistencia a los antibióticos en los últimos años. Estos hallazgos son inquietantes porque los nuevos agentes antibacterianos desarrollados, como el doripenem, el ceftobiprol y la ceftarolina, no muestran actividad contra *A. baumannii* resistente a cefalosporinas y carbapenémicos ^40^. Boo *et al.*^41^, plantean la posibilidad de que se produzca una co-selección de cepas resistentes a carbapenémicos mediante la adquisición de carbapenemasas de tipo OXA de clase D. Nuestro estudio demostró que todos los aislamientos de *A. baumannii* presentaban la capacidad de hidrolizar cefalosporinas de amplio espectro (ceftazidima y cefepima) y carbapenémicos (imipenem y meropenem). Detectamos la presencia de beta-lactamasa tipo OXA-51 y OXA-58, lo que explicaría la resistencia tan marcada al carbapenem, reportado de manera similar por Gales *et al* ^42^.

Este estudio demostró la escasa capacidad de identificación de especies de *Acinetobacter* por el sistema Vitek-2 GNI. Resalta la necesidad de considerar tales resultados como datos preliminares. La identificación precisa mediante métodos moleculares no solo es importante en la investigación de los brotes causados ​​por especies de *Acinetobacter*, sino que también es relevante en estudios epidemiológicos como este informe. Nuestros resultados son similares a los informes publicados en otras regiones geográficas ^43^ y coinciden con los informes de Karageorgopoulos *et al.*^44^, que encontró sensibilidad a la tigeciclina inferior al 90% de los aislamientos de *Acinetobacter spp*. Lo anterior, lo convierte en una opción de tratamiento potencialmente útil contra bacterias altamente resistentes; sin embargo, algunas de estas bacterias también presentan resistencia a la tigeciclina.

Dentro de aislamientos multirresistentes del complejo ACB y los de *A. baumannii*, se detectó *bla* (TEM-1, CTX-9, OXA-58, IMP-1 y VIM-2). Aunque la presencia de la beta-lactamasa TEM-1 se informa como una de las principales causas de resistencia a los antibióticos beta-lactámicos en los aislados de *A. baumannii*, en este estudio, solo el 22.7% de los aislados fueron portadores del gen *bla*TEM- 1. En los últimos años, la tendencia ha cambiado; el aumento de la resistencia a los carbapenémicos se debe principalmente a la presencia de metalo-beta-lactamasas y carbapenemasa tipo OXA de clase D, cuya sobreexpresión está regulada por la presencia de elementos de inserción aguas arriba, como ISAba1 ^11^.

En este estudio, el 38.5% de los aislados de complejo ACB y el 50.0% de *A. baumannii* presentaron beta-lactamasas tipo OXA-58. A pesar de eso, algunos informes han sugerido que los genes de beta-lactamasas tipo OXA se transportan en integrones ^37^^,^^38^, nuestros resultados mostraron que el 82% de los aislamientos del complejo ACB y *A. baumannii* que contenían estos genes no mostraron relación con integrones. Estos resultados son consistentes con los informados por Poirel *et al.*^45^, quien dijo que estos genes no se encuentran generalmente en forma de casetes de genes y, según el mismo autor, estos genes se transportan en plásmidos o están asociados con un proceso de recombinación homóloga.

La presencia de integrones (clase 1 y 2) en el 40% de los aislamientos fue estadísticamente significativa; ambas clases de integrones se han descrito entre los miembros del género *Acinetobacter* aislados tanto en entornos clínicos como medioambientales ^14^. Se informa que las cepas epidémicas de *A. baumannii* tienden a contener un mayor número de integrones que no son epidémicos, y afirma que el uso de antibióticos tiene un gran impacto en el desarrollo de la diversidad y el mantenimiento de estas cepas en la UCI ^46^. 

El integrón de clase 1 presenta múltiples casetes, lo que confiere resistencia a varios antibióticos, como un sello fenotípico distintivo en los aislados de *A. baumanni*^13^^-^^15^^,^^30^, lo que explicaría la resistencia a múltiples fármacos detectada en aislamientos del complejo ACB y A*. baumannii.* La integración de estos elementos en el cromosoma bacteriano puede afectar la expresión de genes como *bla*OXA-51, que codifica un Amp c, b, y la carbapenemasa cromosómica. En este estudio, los 22 aislamientos de *A. baumannii* mostraron resistencia a cefalosporinas y carbapenémicos; sin embargo, solo el 25% de ellos detectaron integrones dentro del genoma. Del complejo ACB, en 32 aislamientos no fueron detectados integrones, por lo que la resistencia a múltiples antibióticos en estos aislamientos debe relacionarse con otros mecanismos de resistencia que pueden ser mediados por plásmidos o mediante la reducción de la permeabilidad membrana celular o de la pared ^47^. El mayor número de integrones se detectó en aislamientos obtenidos por rastreo de muestras nasales y heridas quirúrgicas. Estos resultados no concuerdan con los obtenidos por Koleman *et al.*^29^, mientras que el mayor número de integrones se detectó en aislados de sangre y secreciones; esta discrepancia probablemente se deba a la mayor cantidad de aislados de este estudio que se obtuvieron a partir de la colonización nasal y la ausencia de infecciones.
